# A Statistical Approach to the Diagnosis and Prediction of HCC Using CK19 and Glypican 3 Biomarkers

**DOI:** 10.3390/diagnostics13071253

**Published:** 2023-03-27

**Authors:** Călin Burciu, Roxana Șirli, Renata Bende, Alexandru Popa, Deiana Vuletici, Bogdan Miuțescu, Iulia Rațiu, Alina Popescu, Ioan Sporea, Mirela Dănilă

**Affiliations:** 1 Department of Gastroenterology and Hepatology, “Victor Babes” University of Medicine and Pharmacy, 300041 Timișoara, Romania; 2 Advanced Regional Research Center in Gastroenterology and Hepatology, “Victor Babes” University of Medicine and Pharmacy, 30041 Timisoara, Romania

**Keywords:** cytokeratin 19, glypican-3, hepatocellular carcinoma, statistical model

## Abstract

Various statistical models predict the probability of developing hepatocellular carcinoma (HCC) in patients with cirrhosis, with GALAD being one of the most extensively studied scores. Biomarkers like alpha-fetoprotein (AFP), AFP-L3, and des-g-carboxyprothrombin (DCP) are widely used alone or in conjunction with ultrasound to screen for HCC. Our study aimed to compare the effectiveness of Cytokeratin 19 (CK19) and Glypican-3 (GPC3) as standalone biomarkers and in a statistical model to predict the likelihood of HCC. We conducted a monocentric prospective study involving 154 participants with previously diagnosed liver cirrhosis, divided into two groups: 95 patients with confirmed HCC based on clinical, biological, and imaging features and 59 patients without HCC. We measured the levels of AFP, AFP-L3, DCP, GPC3, and CK19 in both groups. We used univariate and multivariate statistical analyses to evaluate the ability of GPC3 and CK19 to predict the presence of HCC and incorporated them into a statistical model—the GALKA score—which was then compared to the GALAD score. AFP performed better than AFP-F3, DCP, GPC3, and CK19 in predicting the presence of HCC in our cohort. Additionally, GPC3 outperformed CK19. We used multivariate analysis to compute the GALKA score to predict the presence of HCC. Using these predictors, the following score was formulated: 0.005*AFP-L3 + 0.00069*AFP + 0.000066*GPC3 + 0.01*CK19 + 0.235*Serum Albumin—0.277. The optimal cutoff was >0.32 (AUROC = 0.98, sensitivity: 96.8%, specificity: 93%, positive predictive value—95.8%, negative predictive value—94.8%). The GALKA score had a similar predictive value to the GALAD score for the presence of HCC. In conclusion, AFP, AFP-L3, and DCP were the best biomarkers for predicting the likelihood of HCC. Our score performed well overall and was comparable to the GALAD score.

## 1. Introduction

Liver cancer is a significant global health concern, causing 830,000 deaths annually [1]. It is the fifth most common cancer, with 854,000 new cases yearly [2]. Hepatocellular carcinoma (HCC) represents over 90% of primary liver malignant tumors. Approximately 75% of all worldwide diagnosed malignant liver tumors occur in Asia, with China accounting for 50% of the global total [3]. The etiology is different around the world; in eastern Europe, the leading causes are alcohol (53%) and hepatitis C virus (HCV) (24%) [4].

Cirrhosis is a significant risk factor for HCC [4]; the risk of developing HCC for patients with cirrhosis ranges from 1 to 8% per year [5], and approximately one-third of cirrhotic patients will develop HCC during their lifetime [6]. One-quarter of cirrhosis patients die of HCC because tumors are frequently detected at an advanced stage when treatment options are limited [7]. In addition to prevention, early detection could be the key to an increased survival rate. Numerous guidelines define the risk groups and the tools for early detection of HCC. Despite its low sensitivity and specificity, an ultrasound examination performed by experienced personnel is a valuable tool for surveillance, as recommended by American, Asian, and European guidelines [4,8,9].

The cost-efficiency of using biomarkers to screen patients at risk of developing HCC is a topic of debate [10,11]. Multiple biomarkers have been studied for their potential role in the surveillance and early detection of HCC, alongside new prognostic or therapeutic targets for HCC [12,13]. Among them, alpha-fetoprotein (AFP) is the most extensively studied, and guidelines recommend it be used with ultrasound to screen patients at risk for HCC [6]. Despite its widespread use, AFP’s diagnostic accuracy for small HCC is limited [14]. Additionally, AFP levels can be affected by other factors, such as chronic viral hepatitis, gonadal malignancy, and pregnancy, which can lead to variations in its performance as a biomarker [15,16]. The lens culinaris agglutinin-reactive fraction of alpha-fetoprotein (AFP-L3) is an isoform of AFP that exhibits modifications in its carbohydrate chain. AFP-L3 is considered a more specific biomarker for diagnosing HCC than AFP [17,18,19]. Moreover, AFP-L3 > 10% can be a preoperative indicator of anatomic resection for HCC [20].

Des-gamma-carboxyprothrombin (DCP), also known as prothrombin induced by vitamin K absence-II (PIVKA-II), is a highly specific marker for HCC, a predictor of the evolution of HCC patients, and has a higher diagnostic performance than AFP [21]. Glypican-3 (GPC3) was demonstrated to be a biomarker with higher expression than AFP in patients with HCC [22]. The advantage is that it has not been detected in the normal liver or benign liver lesions [23], and it has also not been found in patients with hepatitis [24]. The optimal performance of GPC3 is achieved when combined with other biomarkers: HSP70 (heat shock protein 70) + GS (glutamine synthetase) + GPC3, allowing the differentiation between early-grade HCC and dysplastic nodules arising in cirrhosis [25]. GPC3 can also be used as a survival marker; GPC3-positive HCC patients have a lower 5-year survival rate. [26]. Cytokeratin 19 (CK19) is a biliary cell marker; approximately 30% of patients with HCC express this cytokeratin [27]. CK19 has been associated with HCC and cholangiocarcinoma (CC) pathological features, which are correlated with aggressive behaviors and a poorer prognosis [28].

However, all single serum markers for early surveillance of HCC have yet to be proven to meet the clinical demands [4,8,9]. In the conditions in which previous studies showed that the combination of several biomarkers for the diagnosis of HCC is superior to a single biomarker [25], the next step was the creation of statistical models that combine biological parameters and different anthropometric components [29].

The GALAD score, which combines gender, age, AFP-L3, AFP, and DCP [29], has demonstrated high accuracy in detecting early-stage HCC in patients with cirrhosis [30]. While there is some debate regarding the utility of the GALAD score for HCC screening [31], studies have shown that combining the GALAD score with ultrasound can result in superior detection rates of HCC compared to ultrasound alone [32].

Our study aimed to assess and compare the diagnostic performance of five biomarkers (AFP, AFP-L3, DCP, CK19, and GPC3) for predicting the likelihood of HCC in a cohort of patients from Western Romania. The performance of each biomarker and their combined performance in a statistical model for predicting the likelihood of HCC were evaluated. Additionally, the performance of our model was compared with the GALAD score. To our knowledge, this is the first study of this type in which these five biomarkers are evaluated in the same group of patients and CK19 and GP3 are integrated into a statistical model.

## 2. Materials and Methods

### 2.1. Patients

A monocentric, prospective study was conducted, and participants were divided into two groups. The first group consisted of 95 patients with both HCC and liver cirrhosis, while the second group consisted of 59 patients with liver cirrhosis but without HCC. The presence of HCC in the control group was ruled out using ultrasound performed by an expert in the field. In cases where there was a suspicion of HCC, further diagnostic tests such as contrast-enhanced ultrasound (CEUS), contrast-enhanced computed tomography (CE-CT), or contrast-enhanced magnetic resonance imaging (CE-MRI) were performed.

The inclusion criteria for the HCC and control groups included a positive diagnosis of cirrhosis, signed informed consent, and being over 18 years old. In addition, patients with HCC should be newly diagnosed, whether palliative or curative, without any specific treatment for HCC. An exclusion criterion for both groups was the use of oral anticoagulants.

The diagnosis of liver cirrhosis was established based on clinical signs, liver elastography (FibroScan^®^, Echosens^TM^, Paris, France), and/or biological scores (FibroTest-ActiTest, FibroMax). HCC diagnosis was established based on the European Association for the Study of the Liver (EASL) guideline [4]: a focal liver lesion showing hypervascularity in the arterial phase and washout on the portal venous and/or delayed phases using CEUS, CE-CT, or CE-MRI [9]. The Barcelona Clinic Liver Cancer (BCLC) staging method was used to classify HCC [9].

All patients underwent a clinical examination, a comprehensive medical history was taken, and liver function tests were performed, including alanine aminotransferase (ALT), aspartate aminotransferase (AST), platelet count, albumin, total bilirubin (TB) levels, direct bilirubin (DB), alkaline phosphatase (ALP), and gamma-glutamyl transferase (GGT). Additionally, we measured the levels of AFP, AFP-L3, DCP, GPC3, and CK19 in both study groups. The GALAD score was calculated according to the following formula: Z = 10.08 + 0.09 × age + 1.67 × sex + 2.34 × log10(AFP) + 0.04 × AFP-L3 + 1.33 × log10(DCP), where sex = 1 for males and 0 for females [29].

### 2.2. Biomarkers Assessment

Serum samples were collected by obtaining 10 mL of whole blood using either EDTA or heparin as an anticoagulant. After centrifugation for 15 min at 1000× *g* at 2–8 °C within 30 min, serum samples were collected. To prevent repeated freeze-thaw cycles, serum samples were stored at −80 °C until biomarkers were assessed.

All five biomarkers were quantified using ELISA 96-well strip plate kits, Cusabio^®^ (Houston, TX, USA) for DCP and AFP-L3, R&D Systems^®^ (Minneapolis, MN, USA) for AFP and GPC3, ABclonal^®^ (Wuhan, China) for CK19. The GloMax^®^ Discover Microplate Reader (Promega, Madison, WI, USA) was used to interpret the plates.

### 2.3. Statistical Method

MedCalc Version 19.4 (MedCalc Software Corp., Brunswick, ME, USA) and Microsoft Office Excel 2019 (Microsoft for Windows, Redmond, WA, USA) were used for the statistical analysis, while the demographic, anthropometric, and clinical data of patients’ descriptive statistics were used. The distribution of numerical variables was evaluated using the Kolmogorov–Smirnov test, and continuous numerical variables with normal distributions were presented as means with standard deviations (SDs); in the case of variables with non-normal distributions, we used the median and interquartile ranges (IQRs); the categorical variables were communicated as frequencies and percentages. The Student’s *t*-test was utilized for group comparisons of continuous variables with normal distribution; for variables with non-normal distribution, we used a Mann–Whitney U-test. A *p*-value lower than 0.05 was considered significant for all statistical analyses.

Cutoffs found in papers with similar objectives were utilized to evaluate the biomarkers. For AFP, a cutoff of 20 ng/dL was applied, as it had been found to strike a balance between sensitivity and specificity in previous studies [33,34]. For AFP-L3, a cutoff value of 7% was used. It was discovered by Tamura et al. that this cutoff value best distinguishes HCC from benign liver disease [35], which was later confirmed in another paper [36]. A 40 ng/mL cutoff for DCP was used, consistent with another report that validated the GALAD model for HCC detection in Chinese patients [37]. Due to the limited number of studies on CK19, the 6.25 ng/mL cutoff proposed by El Raziky et al. was adopted [38]. Similarly, for GP3, studies that employed the same quantification method were identified, and the 0.0414 ng/mL cutoff suggested by Liu et al. was utilized [39].

Areas under receiver operating characteristic curves (AUROC) were calculated to identify discriminating cut-off values. The optimal cut-off values were calculated from the AUROC curve analysis using the Bayesian analysis, the optimal criterion (the cut-off value with the highest sum of Se and Sp), and avoiding the misclassification of true positive subjects. Positive predictive value (PPV, defined as the ratio between the true positive cases and all the positive cases), negative predictive value (NPV, defined as the ratio between the true negative cases and all the negative cases), and diagnostic accuracy (defined as the ratio between the sum of the true positive cases and the true negative cases and the total number of cases) were calculated. 95% confidence intervals (CI) were determined for each predictive test, and a value below 0.05 was considered to concede statistical significance. Univariate and multivariate regression analyses were used to find the main independent factors associated with the presence of HCC. The multivariate regression model was built using the Akaike criterion to assess the impact of several factors on the variance of continuous variables. The model was validated based on the accuracy of prediction and R squared. In the final regression equations, the predictors were accepted according to a repeated backward-stepwise algorithm (inclusion criteria *p* < 0.05, exclusion criteria *p* > 0.10) to obtain the most appropriate theoretical model to fit the collected data.

## 3. Results

### 3.1. Demographic and Clinical Characteristics of Patients

**Figure 1:**
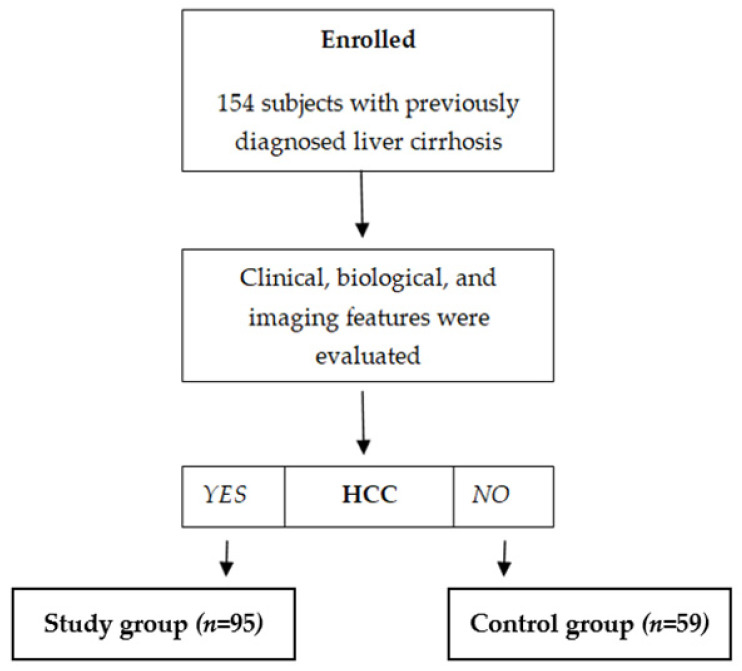
Enrollment of subjects.

One hundred fifty-four subjects with previously diagnosed liver cirrhosis were included, and 95/154 (61.7%) were diagnosed with HCC based on clinical, biological, and imaging features. According to the presence of HCC, subjects were divided into two distinct subgroups: a subgroup of subjects with liver cirrhosis and HCC (*n* = 95) and a control subgroup with subjects previously diagnosed with liver cirrhosis but without HCC (*n* = 59) (Figure 1). The demographic and clinical characteristics of the patients are summarized in Table 1, and the laboratory results are in Table 2.

A more detailed analysis was conducted on GPC3 values based on gender. In patients with HCC, no significant differences were observed between the median GPC3 values for males and females (male GPC3 median values: 424.24 [150.45–6422.11] vs. female GPC3 median values: 373.66 [129.48–10032.18], *p* = 0.6770). However, in patients without HCC, GPC3 median values were notably higher in females compared to males (364.57 [170.93–611.17] vs. 219.56 [29.75–699.96], *p* = 0.0027).

### 3.2. Diagnostic Role of AFP, AFP-F3, DCP, Glypican, and CK-19 Levels

The median values of AFP, AFP-F3, DCP, GPC3, and CK-19 levels for all the included subjects (*n* = 154) were as follows: 31.06 [0.25–1423], 2.68 [0.09–98.1], 1.49 [0.1–1061], 319.15 [29.75–10032.18], and 1.31 [0.13–41.91], respectively. Significant differences were found between these factors in subjects with HCC compared to those without (*p* < 0.0001), except for CK-19 (*p* = 0.0763) (Table 3).

Using the following cut-off values for the subjects with HCC (*n* = 95): 20 ng/dL for AFP, 7% for AFP-L3, 40 ng/mL for DCP, 0.0414 ng/mL for GPC, and 6.25 ng/mL for CK19, we found out that 80/95 (84.2%) patients had elevated AFP, 66 (69.5%) patients had elevated AFP-F3, and 94 (98.5%) patients had elevated DCP. All three tumor markers were elevated in 52 (54.7%) patients.

In a more comprehensive analysis, the 15 patients with HCC who had AFP values below the cut-off value (20 ng/dL) were categorized based on their DCP levels. Among these 15 HCC patients with low AFP, 12 had high DCP levels (>40 ng/mL), thereby increasing the proportion of patients accurately identified as having HCC from 84.2% to 96.8%. When CK-19 and GPC3 values were used together, the proportion of patients correctly classified as having HCC rose from 84.2% to 92.6%.

**Figure 2:**
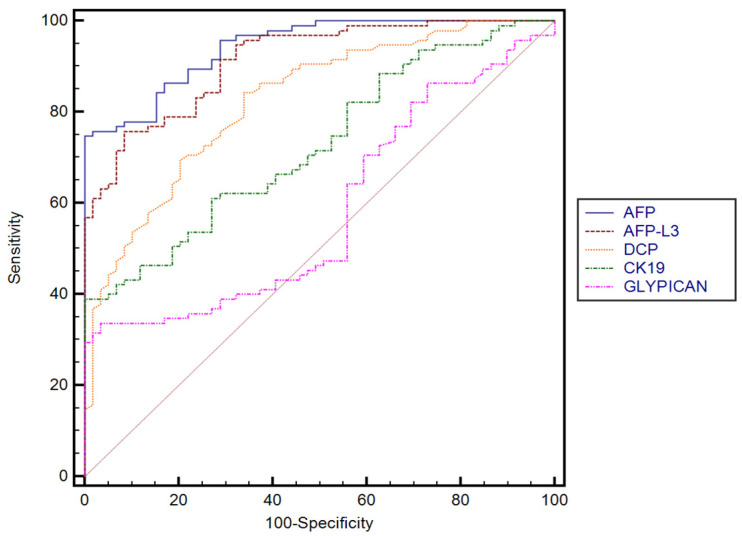
Comparison between receiver operating characteristics for different serum biomarkers.

The performance of AFP, AFP-F3, DCP, GPC3, and CK-19 for predicting the presence of HCC established in our cohort, with their corresponding sensitivities and specificities, is summarized in Table 3. DCP performed better than GPC3 (*p* < 0.0001) and CK-19 (*p* < 0.0001). No significant differences were found between AFP and AFP-L3, while both AFP and AFP-L3 performed better than GPC3 (*p* < 0.0001) and CK-19 (*p* < 0.0001). Furthermore, GPC3 performed better in predicting the presence of HCC than CK-19 (*p* = 0.0067) (Figure 2).

### 3.3. GALAD and GALKA Scores for Predicting the Presence of HCC

**Figure 3:**
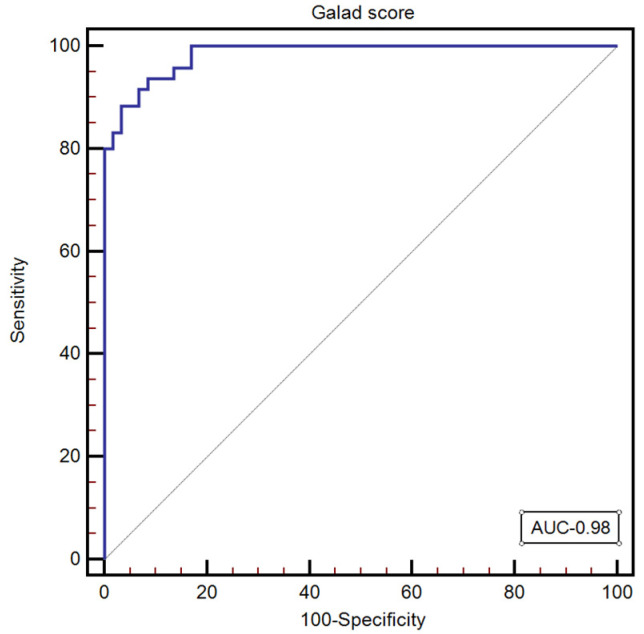
The performance of the GALAD score for predicting the presence of HCC.

GALAD score was calculated for all the subjects included in our study, with a median value of—1.34 [−5.08–7.26]. The optimal GALAD score cut-off value for predicting the presence of HCC was >0.19 (AUC: 0.98, 95% CI: 0.949–0.997; Se: 93.7; Sp: 91.5; NPV: 90.1; PPV: 94.7) (Figure 3).

Univariate and multivariate regression analyses were employed to develop a new prediction score using the previously mentioned markers.

In the univariate analysis, we observed a significant difference between patients with or without HCC regarding the following parameters: age (*p* < 0.001), ALT (*p* < 0.001), AFP (*p* < 0.001), AFP-L3 (*p* < 0.001), DCP (*p* < 0.001), GPC3 (*p* < 0.001), CK-19 (*p* < 0.001), serum bilirubin levels (*p* = 0.001), and serum albumin levels (*p* = 0.001). Multivariate regression analysis was used to identify factors associated with HCC and used a significance level of 0.05. Three regression models were employed (Table 4). The regression model was built based on the stepwise forward method, and Akaike information criteria (AIC) were used to evaluate the best model.

The model including AFP (*p* = 0.0001, β = 0.00069 ± 0.00016, OR = 0.985 [0.973–0.997])), AFP-L3 (*p* = 0.0031, β = 0.005 ± 0.0016, OR = 1.084 [1.008–1.166]), GPC3 (*p* = 0.0013, β = 0.000066 ± 0.000020, OR = 0.924 [0.913–0.961]), CK-19 (*p* = 0.038, β = 0.01 ± 0.005, OR = 0.942 [0.924–0.976]), and serum albumin levels (*p* < 0.0001, β = 0.235 ± 0.041) was the best model for predicting the presence of HCC (*p* < 0.001).

Using these predictors, the GALKA score was formulated: 0.005*AFP-L3 + 0.00069*AFP + 0.000066*GPC3 + 0.01*CK19 + 0.235*serum albumin—0.277. The score was calculated for all the included subjects, and the optimal cutoff value for predicting HCC was >0.32 (AUROC = 0.98, Se-96.8%, Sp-93%, PPV-95.8%, NPV-94.8%).

**Figure 4:**
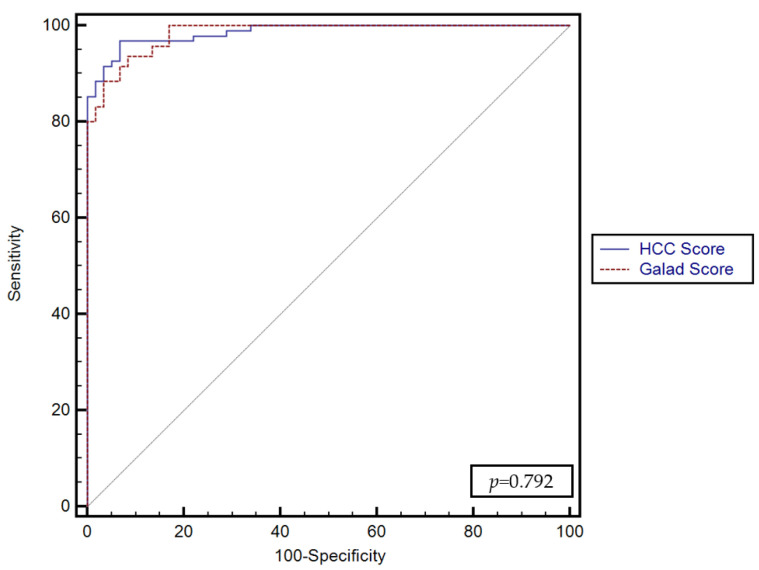
Comparison between the performance of the GALKA and GALAD scores for predicting the presence of HCC.

No significant differences were found between the predictive performance of the GALKA score proposed by our study and the GALAD score (*p* = 0.792) (Figure 4).

These scores were also tested for predicting the presence of portal vein thrombosis. No significant differences were found between their predictive performance (AUC: 0.67, 95% CI: 0.593–0.746 for GALAD vs. AUC: 0.63, 95% CI: 0.553–0.710 for GALKA, *p* = 0.4918), while GALAD score performed better for predicting the size of the HCC (>5 cm) (AUC: 0.78, 95% CI: 0.716–0.850) compared to GALKA score (AUC: 0.69, 95% CI: 0.619–0.770), *p* = 0.0069.

## 4. Discussion

HCC is a significant health problem due to its high mortality rate [1]. As with other neoplastic diseases, early diagnosis is crucial in addressing this issue, which can be achieved through a sustainable screening program. The EASL [4] and the American Association for the Study of Liver Diseases (AASLD) [9] guidelines recommend an ultrasound examination every six months, with or without AFP, to screen for HCC. Conversely, the Asian [8,40] guidelines suggest the use of biomarkers. There is considerable disagreement in the scientific and medical communities regarding the use of blood biomarkers for monitoring patients at risk of developing HCC. The problems are related to various sensitivities and specificities among studies, the heterogeneity of groups, and various cutoffs. In our study, we evaluated five biomarkers from the same sample and their ability to diagnose HCC.

In our study, AFP levels in the HCC group were significantly higher than in the cirrhosis group (<0.0001). These results are consistent with other studies [41,42]. However, the cutoff value used affects sensitivity and specificity. In our study, using a cutoff of 20 ng/mL for HCC diagnosis, we obtained a sensitivity of 74.7% and a specificity of 100% for AFP. AFP also had the highest AUC (0.94) among the five biomarkers tested. Marrero et al. reported similar sensitivity (59%) and specificity (90%) using a cutoff of 20 ng/mL, which is consistent with our findings [33]. In a recently published meta-analysis, AFP’s pooled sensitivity and specificity were 61% and 87%, respectively [34]. Using a higher cutoff is associated with a decrease in sensitivity, such as 22% for 200 ng/mL [43] and 18% for 400 ng/mL [44]. The excellent specificity of AFP may be due to the fact that over half of HCC patients have a lesion larger than 5 cm or multiple nodules. However, low-AFP HCC patients can still develop large HCCs, indicating that variables other than AFP play a role in determining HCC size [45]. Pang et al. suggested combining AFP with other biomarkers to improve diagnostic efficiency [34]. In our study, adding DCP to evaluate patients with normal/low levels of AFP increased the correct classification rate to 96.8%, while adding CK-19 levels and Glypican increased the correct classification rate to 92.6%.

Consequently, ongoing efforts are being made to identify novel blood biomarkers for HCC, with several new ones having been discovered in recent decades. However, only a few have been adopted in clinical practice—those that are less invasive, easy to replicate, and produce highly consistent results.

In a meta-analysis of six articles with 2447 patients, AFP-L3 was found to have a specificity of 92% and sensitivity of 34%, with an AUC of 0.75 for diagnosing early HCC [46]. In our study, we observed that the specificity of AFP-L3 was 91.5%, the sensitivity was 75.8%, and the AUC was 0.91, suggesting that its sensitivity increases with the larger size and a more advanced stage of HCC. Although AFP-L3 is not very sensitive for early HCC, it has a high specificity that cannot be matched by total AFP and has the added benefit of distinguishing HCC from benign liver disorders in individuals with increased serum AFP [46]. AFP-L3 was also strongly associated with complications of HCC, such as portal vein invasion and intrahepatic metastasis [47].

DCP is another serum biomarker studied for its utility in diagnosing HCC. In our study, DCP had the third-highest AUC at 0.82, performing worse than AFP and AFP-L3. Marrero et al. obtained a similar AUC of 0.72, concluding that AFP was more sensitive than DCP and AFP-L3% [41]. However, DCP has been demonstrated to have a low sensitivity in detecting preclinical HCC, only 26.3% [36]. Compared to these data, in our study, the sensitivity was higher, and we believe that the advanced stage and larger size of the HCC were determinants of this high sensitivity. DCP performs better when combined with other biomarkers, as shown in a phase II study [48] and also observed in our study.

GPC3 is a member of the glypican family of glycosylphosphatidylinositol-anchored cell-surface heparan sulfate proteoglycans. In our study, GPC3 performed better only when compared with CK-19 and was less accurate in diagnosing HCC than AFP, with an AUC of 0.72 vs. 0.94. This inferiority of GPC3 contradicts previously reported data where it was superior to AFP, with sensitivity and specificity of 84–85% and 92–95% vs. 50–79% and 80–90% [22]. One possible explanation for the results may be that GPC3 is more effective than AFP in detecting early-stage liver cancer and is not correlated with tumor size [39]. While the specificity of GPC3 was found to be very good in our study, the relatively low sensitivity observed compared to other studies [22,39] could be attributed to the measurement method used [22] as well as the size of the sample batch [39]. Further research may be needed to validate these findings and to explore other factors that may contribute to the sensitivity of GPC3 as a biomarker for liver cancer. The combination of AFP and GPC3 seems warranted for achieving high accuracy. [39].

GPC3 expression may be higher in females than men since the GPC3 gene is X-linked and situated in the Xq26 region [49]. Interestingly, in subjects without HCC, GPC3 median values were significantly higher in females than males, a phenomenon explained by the fact that about 25% of X-linked genes may escape X chromosome inactivation to some extent. Further studies are needed to investigate the impact of gender on GPC3 levels and, thus, the potential need for sex-specific cutoffs.

CK19 is an HCC stem cell marker involved in carcinogenesis, metastasis, and recurrence [50]. Our study showed no significant difference in serum CK19 levels among patients with and without HCC. Moreover, CK19 performed less accurately in diagnosing HCC than all other biomarkers in our study. Similar data were reported by Raziky et al., with reported sensitivity and specificity of 63.4% and 55%, respectively, who concluded that combining AFP with CK19 offers high sensitivity [38]. When using the combination in our study, we obtained better specificity but with a loss of sensitivity.

Published data showed that using a combination of biomarkers could lead to better detection of HCC. The GALAD (gender, age, AFP-L3, AFP, and DCP) score combines serum-based markers (AFP, AFP-L3, and DCP) with demographic information (gender and age) [51]. Therefore, we evaluated the performance of the GALAD model for diagnosing HCC in our group. Our results showed that GALAD had high accuracy for HCC detection, with an AUC of 0.98. We used GALAD’s concepts to create a new HCC diagnostic model—the GALKA score. The model included AFP, AFP-L3, Glypican, CK19, and serum albumin levels, variables that showed the best model for predicting the presence of HCC (*p* < 0.001). Compared with GALAD, we added serum albumin to our multivariate logistic regression analysis, an independent factor associated with developing HCC that reflects synthetic liver function.

In our current study, the GALKA score model had a higher AUC (0.98) and a higher sensitivity and specificity in diagnosing HCC compared to other biomarkers evaluated. Compared with GALAD, GALKA had a similar AUC; however, the sensitivity and specificity were higher, at 96.8% and 93% vs. 93.7% and 91.5%, respectively.

Combining four HCC-specific biomarkers and one protein reflecting liver function has a synergistic effect, improving overall diagnostic accuracy. Furthermore, the absence of DCP from our score eliminates interference with anticoagulants when applying the score in the real world.

In a recent paper, Li et al. validated the GALAD score and compared it with other scores and biomarker combinations, demonstrating the superiority of GALAD (AUC 0.925, 0.945) [37]. Furthermore, a systematic review published by Guan et al. supports the robust power of GALAD as an HCC screening or diagnostic tool [52]. However, a phase 3 biomarker study from the United States showed that GALAD’s performance was modest and not different from AFP-L3 alone or carcinoma early detection screening (HES) [53]. GALAD’s performance is also influenced by the etiology of the chronic liver disease of the patient at high risk for HCC, with decreased performance in HBV etiology and higher pooled sensitivities and AUC values seen in HCV and non-viral liver disease patients [52]. However, the number of HCV cirrhotic patients is decreasing due to direct-acting antivirals (DAA), while NAFLD is increasing. Nevertheless, GALAD has a good AUC of 0.91 in detecting NAFLD-associated HCC [54].

GALAD’s sensitivity and AUC are associated with the BCLC stage [52]. Considering that approximately half of our cohort is outside the Milan criteria, this may explain the high AUC and sensitivity of GALAD in our cohort. However, the GALAD score performed better in predicting the size of HCC in our study, leading us to hypothesize that the GALKA score could show different performances in a cohort of patients with early-stage HCC. This hypothesis requires validation in a larger patient cohort with early-stage HCC.

Some limitations need to be acknowledged. Firstly, regarding the sample size, we need a larger cohort of patients with HCC and a larger control group to evaluate our score better. Secondly, a cost-effectiveness analysis should have been performed. Although many new technologies for diagnosing HCC are emerging, they come at a higher cost. Future cost-effectiveness analyses can determine whether our score is cost-efficient for diagnosing HCC.

However, given that other scores perform differently in each etiology of patients at risk of HCC, future studies are necessary to divide patients according to their etiology. In conclusion, for the validation and understanding of the place of our score in the diagnosis of HCC, we need more extensive studies with early-stage HCC divided according to their etiologies.

## 5. Conclusions

AFP has been demonstrated to have the best performance in predicting the likelihood of HCC (AUC 0.94), followed by AFP-L3 and DCP. The integration of GPC3 and CK19 into the GALKA score has also shown an overall strong performance (AUC 0.98), similar to the GALAD score, with no significant differences (*p* = 0.792). However, future studies are needed to validate the GALKA score in cohorts of patients with early HCC and to categorize them according to the etiologies of cirrhosis.

## Figures and Tables

**Table 1:** The demographic and clinical characteristics of the patients.

Parameter	Subjects with Hepatocellular Carcinoma	Subjects without Hepatocellular Carcinoma	*p*-Value
	*n* = 95	*n* = 59	
Age (years)	67.27 ± 9.31	60.98 ± 9.77	*p* = 0.001
Gender (%)			
Females	35 (36.8%)	18 (30.5%)	0.532
Males	60 (63.2%)	41 (69.5%)	0.532
Etiology (%)			
ALD	19 (20%)	34 (57.5%)	<0.001
HBV	21 (22.1%)	3 (5.1%)	0.009
HCV	46 (48.4%)	4 (6.8%)	<0.001
HBV + HDV	4 (4.2%)	4 (6.8%)	0.739
HBV + HCV	1 (1.1%)	0 (0%)	0.841
Viral + ALD	0 (0%)	5 (8.5%)	0.015
NASH	4 (4.2%)	5 (8.5%)	0.452
Others	0 (0%)	4 (6.8%)	0.039
Child-Pugh grade (%)			
A [5,6]	39 (41%)	1 (1.7%)	<0.001
B [7–9]	30 (31.6%)	19 (32.2%)	0.920
C [10–15]	26 (27.4%)	39 (66.1%)	<0.001
MELD score	13.26 ± 6.48	20.61 ± 6.13	*p* < 0.001
Tumor stage (BCLC)			
A	25 (26.3%)		
B	17 (17.9%)		
C	16 (16.9%)		
D	37 (38.9%)		
Tumor size (cm)			
<5 cm	40 (42.1%)		
>5 cm			
or multiple nodules	55 (57.9%)		
PVT	20 (21%)	1 (1.7%)	*p* < 0.001

Data are presented as a number and percentage, or mean ± standard deviation; *n*—number; HCV—hepatitis C virus; HBV—hepatitis B virus; HDV—hepatitis D virus; ALD—alcohol-related liver disease; NASH—nonalcoholic fatty liver disease; PVT—portal vein thrombosis.

**Table 2:** Laboratory results.

Parameter	Subjects with Hepatocellular Carcinoma	Subjects without Hepatocellular Carcinoma	*p*-Value
	*n* = 95	*n* = 59	
CK-19 (ng/mL)	1.26 [0.13–41.91]	1.43 [0.16–6.43]	0.076
AFP (ng/mL)	134.62 [2.5–1420.0]	2.47 [0.25–32.96]	<0.001
AFP_L3 (ng/mL)	14.28 [0.33–98.1]	0.98 [0.09–30.27]	<0.001
DCP (ng/mL)	3.12 [0.46–1061]	0.25 [0.10–0.95]	<0.001
Glypican (pg/mL)	423.59 [129.48–10,032.18]	270.81 [29.75–699.96]	<0.001
Serum albumin (g/dL)	3 [1.2–4.3]	2.3 [1.3–3.4]	<0.001
ALT (UI/L)	55 [6–419]	32 [12–444]	<0.001
AST (UI/L)	74 [20–687]	71 [36–1186]	0.445
TB (mg/dL)	1.42 [0.40–16.3]	3.4 [0.2–28]	<0.001
DB (mg/dL)	0.54 [0.05–13.5]	2.08 [0.13–18.6]	<0.001
ALP (UI/L)	117 [36–897]	118 [35–360]	0.996
GGT (UI/L)	100 [14–1363]	138 [28–1156]	0.594
Platelet count	121,000 [40,000–590,000]	129,000 [36,000–351,900]	0.872

*n*—number; CK-19—cytokeratin 19; AFP—alpha-fetoprotein; AFP_L3—alpha-fetoprotein L3; DCP—des-g-carboxyprothrombin; ALT—alanine aminotransferase; AST—aspartate aminotransferase; TB—total bilirubin; DB—direct bilirubin; ALP—alkaline phosphatase; GGT—gamma glutamyl transferase.

**Table 3:** Performance of serum biomarkers for predicting the presence of HCC.

Parameter	AUC	SE	95%CI	Se (%)	95%CI	Sp (%)	95%CI	NPV	PPV
AFP (ng/mL)	0.94	0.016	0.894–0.974	74.7	64.8–83.1	100	93.3–100	100	71.1
AFP-L3 (ng/mL)	0.914	0.022	0.858–0.953	75.8	65.9–84.0	91.5	81.3–97.2	93.5	70.1
DCP (ng/mL)	0.824	0.022	0.755–0.881	84.2	75.3–90.9	66.1	52.6–77.9	72.2	80
GPC3 (pg/mL)	0.726	0.039	0.649–0.795	38.95	29.1–49.5	100	93.3–100	100	50.4
CK-19 (ng/mL)	0.585	0.046	0.503–0.664	33.7	24.3–44.1	96.6	88.3–99.6	94.1	47.5

CK-19—cytokeratin 19; AFP—alpha-fetoprotein; AFP-L3—alpha-fetoprotein L3; DCP—des-g-carboxyprothrombin.

**Table 4:** Models’ performance for discriminating HCC (*n* = 80) and CLD (*n* = 139) (specificity = 90%).

Model	AUC	95% CI	Sensitivity
Model 1	0.912	0.889–0.947	90.9%
Model 2	0.931	0.912–0.966	92.0%
Model 3—GALKA	0.981	0.946–0.997	96.8%

Model 1 consists of age (*p* < 0.001), ALT (*p* < 0.001), AFP (*p* < 0.001), AFP-L3 (*p* < 0.001), DCP (*p* < 0.001), GPC3 (*p* < 0.001), CK-19 (*p* < 0.001), serum bilirubin levels (*p* = 0.001); Model 2 consists of ALT (*p* < 0.001), AFP (*p* < 0.001), AFP-L3 (*p* < 0.001), DCP (*p* < 0.001), GPC3 (*p* < 0.001), CK-19 (*p* < 0.001), serum albumin levels, Model 3—GALKA consists of AFP, AFP-L3, GPC3, CK-19, and serum albumin levels.

## Data Availability

Data are available on request.
